# Predictive Factors of Treatment-Resistant Schizophrenia

**DOI:** 10.1192/j.eurpsy.2025.1463

**Published:** 2025-08-26

**Authors:** I. Bouamoud, N. Barkallil, S. Belbachir, A. Ouanass

**Affiliations:** 1 University Psychiatric Hospital Arrazi, Salé, Morocco

## Abstract

**Introduction:**

Schizophrenia is a complex, chronic psychiatric disorder marked by disruptions in thought processes, perception, social interactions, and emotional regulation. Despite various therapeutic options, 20–34% of patients develop treatment-resistant schizophrenia (TRS)¹, a condition associated with a poor prognosis and significant challenges in clinical management. Early identification of predictive factors for treatment resistance may enable more targeted interventions, ultimately improving patient outcomes by allowing for tailored treatment approaches.

**Objectives:**

This study aims to identify early predictive factors for the progression to TRS, differentiate modifiable from non-modifiable factors, and determine prognostic indicators for schizophrenia. The goal is to facilitate early intervention for high-risk cases and prevent TRS by targeting modifiable factors.

**Methods:**

This is a descriptive and an analytical retrospective study including patients diagnosed with TRS according to NICE criteria, treated with Clozapine, and hospitalized at the Ar-Razi Psychiatric Hospital in Salé between 2022 and 2024. Included cases meet specific criteria, such as complete clinical records and the need for Clozapine treatment, with no age restrictions. Sociodemographic, clinical, evolutionary, and therapeutic data are collected using Excel and analyzed using SPSS 20 software.

**Results:**

Among the 126 TRS cases included, several risk factors were identified. Non-modifiable factors include age, family history, and the presence of negative symptoms, while modifiable factors include the duration of untreated illness and certain comorbidities.

**Conclusions:**

The results of this study provide valuable insights into risk factors associated with TRS and guide specific management and prevention strategies for this subgroup of schizophrenia patients.

**Disclosure of Interest:**

None Declared

